# Use of Mendelian Randomization to assess the causal status of modifiable exposures for rheumatic diseases

**DOI:** 10.1016/j.berh.2024.101967

**Published:** 2024-06-30

**Authors:** Sizheng Steven Zhao, Stephen Burgess

**Affiliations:** 1 Centre for Musculoskeletal Research, Division of Musculoskeletal and Dermatological Science, School of Biological Sciences, Faculty of Biological Medicine and Health, University of Manchester, Manchester Academic Health Science Centre, Manchester, UK; 2 MRC Biostatistics Unit, University of Cambridge, Cambridge, United Kingdom; 3 British Heart Foundation Cardiovascular Epidemiology Unit, Department of Public Health and Primary Care, University of Cambridge, Cambridge, United Kingdom

**Keywords:** instrumental variable, genetics, causal inference, rheumatology, rheumatoid arthritis

## Abstract

The explosion in Mendelian randomization (MR) publications is hard to ignore and shows no signs of slowing. Clinician readers, who may not be familiar with jargon-ridden methods, are expected to discern the good from the many low-quality studies that make overconfident claims of causality or stretch the plausibility of what MR can investigate. We aim to equip readers with foundational concepts, contextualized using examples in rheumatology, to appraise the many MR papers that are or will appear in their journals. We highlight the importance of assessing whether exposures are under plausibly specific genetic influence, whether the hypothesized causal pathways make biological sense, and whether results stand up to replication and use of control outcomes. Quality of research can vary substantially using MR as with any design, and all methods have inherent limitations. MR studies have provided and can still contribute valuable insights in the context of evidence triangulation.

## Introduction

The sheer volume of Mendelian randomization (MR) papers flooding clinical journals makes it hard to ignore. At the current pace of growth, it will (if it has not already) become one of the dominant methods used in observational epidemiological research. Yet, MR can be unfamiliar to many clinicians because instrumental variable approaches are often not taught in undergraduate medical curricula. For instance, where would the reader place MR in the traditional hierarchy of evidence, starting from case reports and going up to systematic reviews of randomized controlled trials? Even for seasoned academics, MR sits at an intersection between traditional epidemiology and genetic disciplines, combining concepts not universally familiar to researchers in either field.

Factors driving the explosion in MR publications, such as publicly available data and software (and flawed incentives to ‘publish or perish’), will not change. As biobanks and genetic studies grow in scale, every conceivable trait will be put through ever larger genome-wide association studies (GWASs) that feed the exponential increase in MR papers, no matter how problematic their genetic underpinning is. The volume of submissions versus a comparatively small pool of experienced reviewers mean that low-quality studies, often stretching the plausibility of what MR can be used to investigate, make it through to publication. Readers are then presented with overconfident claims of causality using jargon-ridden methods that promise robustness against confounding and other biases.

It is in this context that we hope to arm the reader with a foundation to appraise the many MR papers that are or will appear in their journals. We start with the concept of and assumptions required for MR, including examples related to rheumatology to contextualize common issues, and finish with examples of useful applications of the technique.

### What is MR?

**Figure 1:**
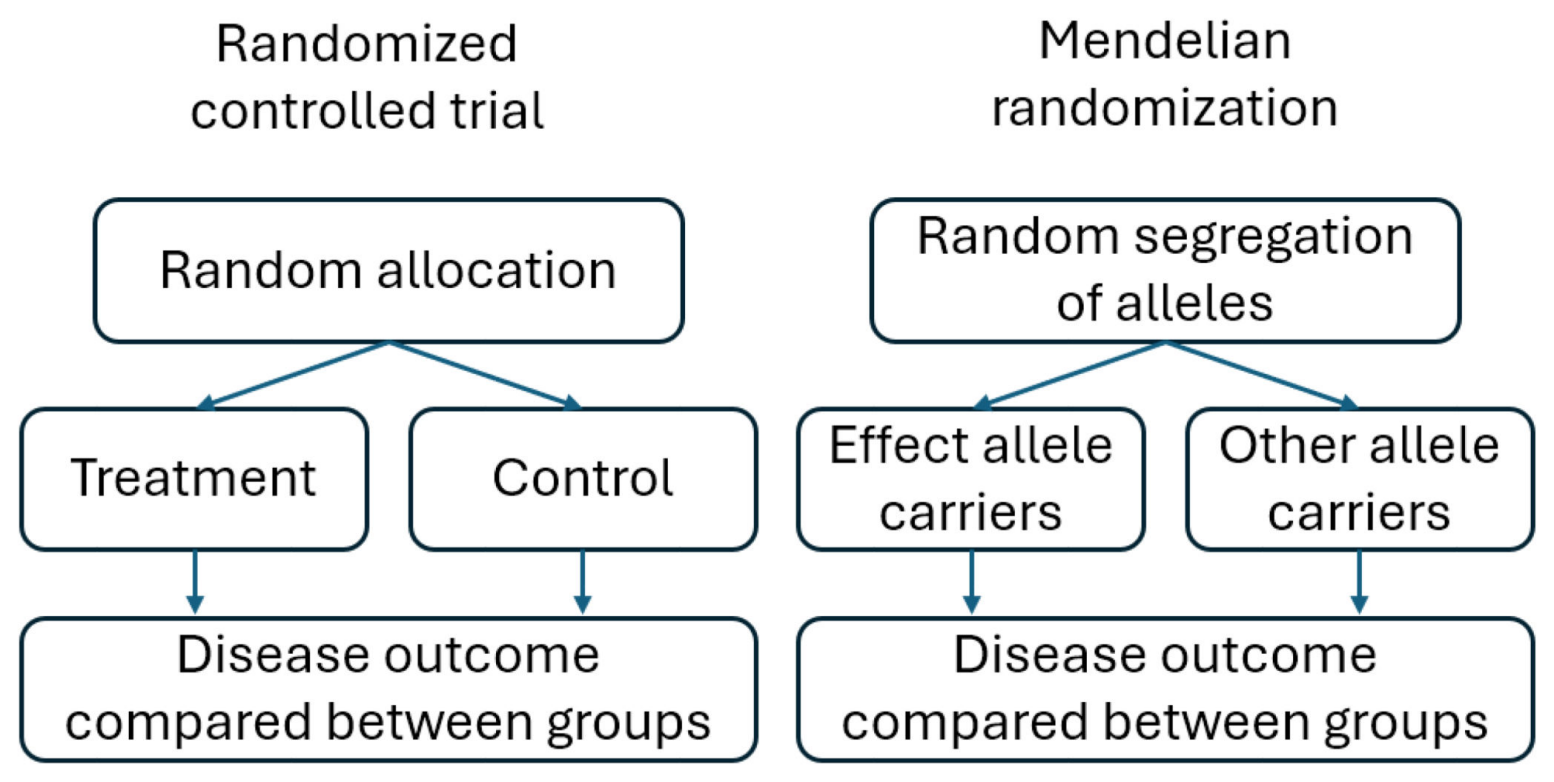
Analogy between a simple Mendelian randomization design and a randomized controlled trial.

Of the many ways to describe instrumental variable approaches, one intuitive explanation is as a natural experiment. In the case of MR, nature in effect conducts randomized experiments, where some individuals inherit genetic variants that influence an exposure, while others do not (Figure 1) [1]. MR and other quasi-randomized methods (e.g., interrupted time series) therefore sit at the interface between randomized and non-randomized (i.e., traditional observational) designs [2]. A list of key terminology used in MR is provided in Table 1.

Consider the question of whether hyperuricaemia increases risk of ischaemic heart disease (IHD). This is a challenging question to answer using traditional observational designs because: first, hyperuricaemia is closely related to other cardiovascular risk factors (e.g., obesity and gout); and second, symptoms or pre-clinical manifestations of heart disease may in turn affect urate levels through dietary or therapeutic changes. These twin concerns for confounding and reverse causation are often invoked as motivations for trusting MR over traditional epidemiologic approaches.

Some individuals inherit a genetic predisposition to hyperuricaemia. Each additional A allele for the *SLC2A9* single nucleotide polymorphism (SNP) rs7442295 is associated with 0.02mmol/L higher serum urate, and 5% higher risk of hyperuricaemia [3]. Such genetic variants can be used to divide the population into groups with differing average levels of serum urate, analogous to arms of a randomized controlled trial. When certain assumptions hold (discussed in the next section), other cardiovascular risk factors are distributed as if randomly across these groups. If the genetically defined groups differ in their incidence of IHD, then under the MR assumptions we can infer that serum urate has a causal effect on IHD risk. Using a traditional observational design, hyperuricaemia was associated with over two-fold increase in IHD risk (potentially explained by residual confounding from BMI), whilst MR did not support a causal relationship between hyperuricaemia and IHD [3].

### What MR can and cannot do

Not all exposures have a plausible genetic basis. Genes encode proteins that in turn influence measurable traits. While genes can have important influences on traits like cytokine levels or exposures metabolised by enzymes, it is less conceivable that exposures based on where people live (e.g., exposure to air pollution, which is typically not measured but estimated based on home address [4]) or specific dietary behaviours (e.g., breakfast skipping [5] or dried fruit intake [6]) are under credibly specific genetic influence. Even if an exposure has genome-wide significant predictors, MR requires genetic variants to be specific in how they influence the exposure. Just because we can find GWAS hits for a given trait does not mean that the trait is a suitable exposure for a MR investigation.

The first question a reader should ask is: Can genetic variation plausibly influence the exposure in ways analogous to a proposed intervention? MR only makes sense when there is gene-environment equivalence. For example, genetic variation in the *SOST* gene that encodes sclerostin leads to low or absent sclerostin levels and high bone mass [7], while therapeutic sclerostin inhibition using romosozumab increases bone mass. There is sufficient analogy between genetic and therapeutic perturbation in sclerostin to allow inferences about drug efficacy and safety to be drawn from genetic associations in this gene region. If genetically predicted sclerostin function is associated with adverse cardiovascular profile, then there is genetic support for the concern over cardiovascular risk when using romosozumab [8,9].

Gene-environment equivalence is an important sense checking step that should first take place, before appraising whether any statistical gymnastics can provide meaningful findings. As shown in the next section, authors can claim various assurances for the instrumental variable assumptions even when it is implausible that genetic variants mimic interventions on the exposure. When biologically plausible genetic variants are thoughtfully selected as instrumental variables, the MR analysis is more likely to fulfil key assumptions required for valid causal inference [10].

### More than just three assumptions

**Figure 2:**
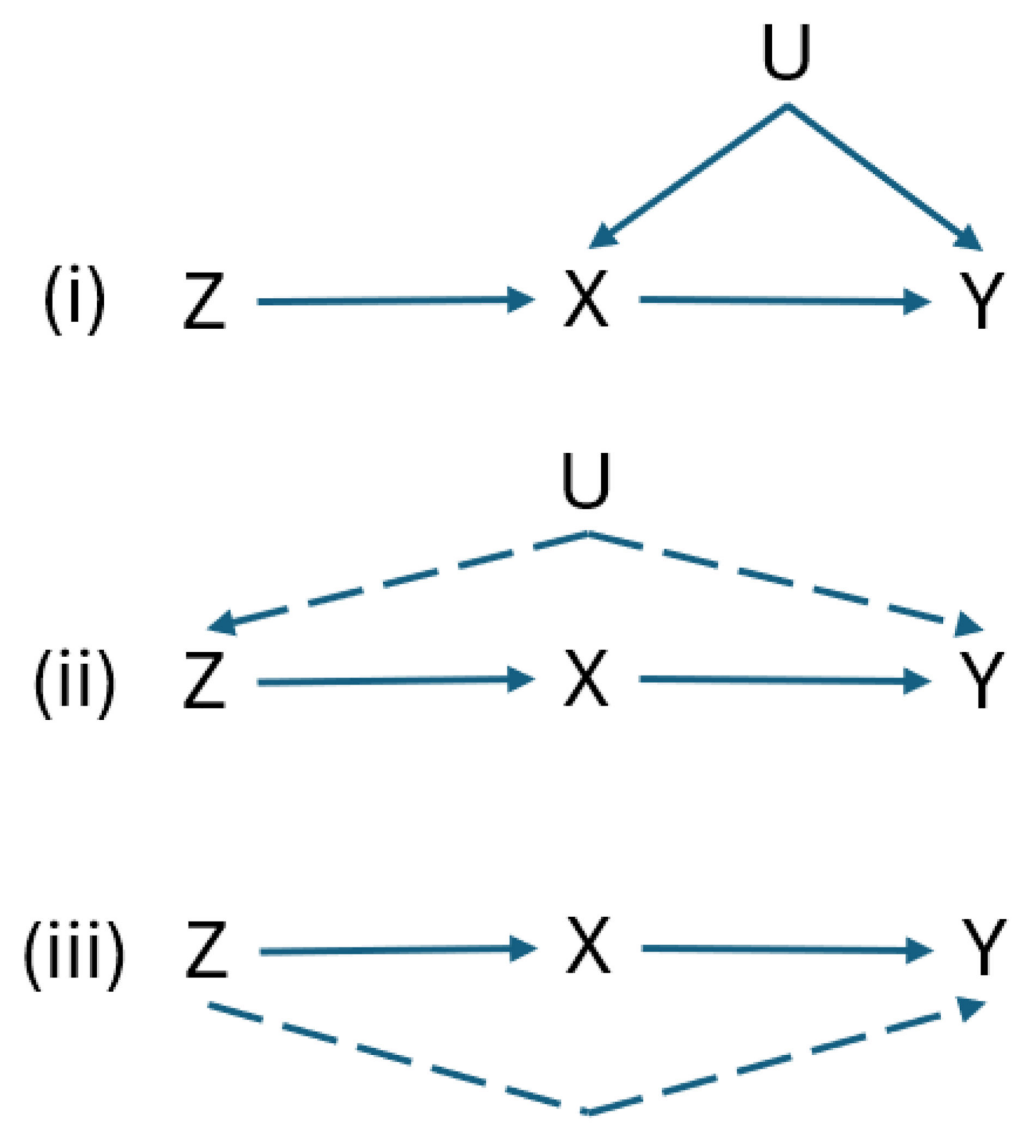
Instrumental variable assumptions required for causal inference in Mendelian randomization. (i) the genetic instrument (Z) is strongly associated with the exposure (X); (ii) no confounders of the instrument (Z) and the outcome (Y); (iii) no causal pathway from the instrument (Z) to the outcome (Y) other than through the exposure (X).

Whether a genetic variant can inform us about the causal status of an exposure depends on three assumptions (Figure 2) referred to as relevance, independence (or exchangeability), and exclusion-restriction (or no horizontal pleiotropy).

First, there must be a robust association between the genetic variant and the exposure. Genetic variants need to separate the population into subgroups that differ in average levels of the exposure to enable comparison. Authors typically present an F-statistic which reflects the extent to which instrumental variables explain variation in the exposure. Genetic instrumental variables are typically said to have sufficient strength if F is greater than 10, although this is a rule-of-thumb to constrain bias rather than a strict threshold above which bias magically vanishes. Having weak instruments does not preclude analysis, but such estimates may be biased towards the confounded exposure-outcome association in one-sample MR (Table 1), and towards the null in two-sample MR (with no sample overlap) [2].

Second, if a factor is associated with the occurrence of both the genetic variant and the outcome (i.e., if the natural experiment is not “as-if randomized”), then the MR estimate may be biased by confounding. For example, such confounding may arise if there is unaccounted structure in the population (such as population stratification, dynastic effects, or assortative mating, summarized in Table 1), or if the selected genetic instrument is correlated (in linkage disequilibrium) with a genetic variant having a pleiotropic effect on a trait on an unrelated causal pathway.

Third, instrumental variables should only affect the outcome through the exposure of interest. The precise function of genetic variants is typically unknown; in the urate example, if the variants in fact have no direct influence on serum urate but instead, say, affect BMI or ischaemic heart disease directly, then we are not actually investigating the effects of urate. This is the most likely assumption to be violated in MR analyses, and the focus of most sensitivity analyses.

There are many other assumptions and considerations for causal inference in MR analyses, for example, selection and survival bias, winner’s curse, use of overlapping datasets, and time-varying effects (see Table 1 and reference [11] for detailed guidance on performing MR investigations). However, it is important to note that authors can present readers with superficially impressive looking manuscripts including statistical assurances for all three assumptions even when the analysis is conceptually problematic. The MR-STROBE checklist [12] was devised to improve the standard of reporting in MR publications. It forces researchers to justify the plausibility of hypotheses, which should reduce more obvious examples of problematic exposures such as estimated exposure to air pollution [4], occupation [13], or specific dietary intakes [5,14]. However, there are seemingly sensible exposures that are nevertheless unsuitable for MR analyses.

### MR for drug prescription or usage

Interstitial lung diseases (ILD) are a group of conditions, some of which can co-occur with rheumatic diseases (e.g., rheumatoid arthritis or systemic sclerosis related ILD) and others without, e.g., idiopathic pulmonary fibrosis (IPF). Statins, which lower cholesterol levels, are hypothesized to have additional anti-fibrotic effects, and observational studies have suggested a protective effect on ILD [15]. It seems, at first glance, reasonable to identify genetic variants strongly associated with use versus non-use of statins to study the effect of statins on IPF risk using MR; specifically, one could select uncorrelated variants associated with statin-use across the genome that reach genome-wide significance.

Readers may believe all three assumptions ‘satisfied’ by a two-sample MR paper that includes large F statistics, ancestrally homogenous populations, and ‘pleiotropy-robust’ methods such as MR-Egger that demonstrate no statistical evidence of directional pleiotropy, and conclude that statin-use affects IPF risk.

However, this analysis should not pass the first sense check, since it is implausible that whether an individual chooses to take a statin is under specific genetic influence. Liability to taking a statin is influenced by having any one of multiple cardiovascular risk factors, clinician/patient preferences, compliance, tolerance, and so forth; genetic variants that associate with increased statin usage may influence any of these traits. Such variants will likely influence multiple traits that violate the third assumption, yet show no statistical evidence of horizontal pleiotropy in sensitivity analyses [16]. Indeed, genetic variants in the *PCSK9* gene region that are associated with increased statin usage, are associated with increased levels of LDL-cholesterol, not reduced levels. This is because the increased statin usage is in response to raised cholesterol levels, and not because statins raise cholesterol [17].

Note that statin-use (assessed in pharmacoepidemiology studies of RCTs) is different from the biological action of statins (assessed using MR). Several genetic variants in the *HMGCR* gene (that encodes the target of statins) influence the function of 3-hydroxy-3-methylglutaryl CoA reductase (HMGCR). Unlike statin-use, reduced HMGCR protein function through genetic or therapeutic influence has credible gene-environment equivalence. Indeed, when genetic instruments for HMGCR perturbation are used, or when the association between statin-use and IPF is adjusted for smoking and/or BMI, there are no associations with IPF risk [16]. Analogously flawed hypotheses include whether genetic liability to use methotrexate is associated with dementia [18] or genetic liability to metformin-use with osteoporosis [19]; in these examples, sensitivity analyses may not reveal statistical evidence of pleiotropy.

Readers may wonder how distinction between drug-use and drug-action applies to exposures such as cigarette smoking, alcohol, or coffee intake. Whether individuals choose to indulge in these lifestyle factors can also be subject to complex behavioural factors under non-specific genetic influence. A purely statistical and biology-agnostic approach to selecting SNPs to instrument genetic liability to their intake is indeed susceptible to pleiotropy. However, alcohol metabolism is relatively well-understood, and genetic variation can influence the rate of clearance or, through adverse effects of metabolites, the likelihood of alcohol consumption. For example, carriers of the A allele for the *ADH1B* (alcohol dehydrogenase 1B enzyme) variant rs1229984 consume 17% fewer units of alcohol per week, and have lower prevalence of binge drinking and higher abstention, compared to non-carriers [20]. Genetic variants can also influence nicotine dependence [21] and caffeine metabolism [22] in a specific way. Applications of MR to investigate drug targets should use genetic variants in biologically-relevant gene regions that mimic the intervention of interest, not variants that simply predict drug prescription or drug usage.

### Causal humility

Many MR papers make overconfident claims of causality, on the basis that genetic variants are randomly allocated at conception, and thus supposedly immune from confounding. Specifically, the authors mean that offspring randomly inherit one allele from each parent at each genetic locus, and that alleles are generally unrelated to each other. However, almost all MR papers use population-level data from unrelated individuals where this ‘natural randomization’ can break down. Genetic associations (therefore MR estimates) can be confounded by underlying population structure, even after adjustment for ancestry principal components [23–25]. Such biases are generally more notable for behavioural (e.g., education, depressive symptoms, smoking) than molecular traits (e.g., CRP, lipids) [25]. In some cases, bias due to population structure may be negligible. However, in all cases, it should provide grounds for caution in the reliability of findings.

Another reason for a measured interpretation of MR findings is ‘horizontal pleiotropy’, often simply referred to as pleiotropy. This is distinguished from ‘vertical pleiotropy’, which occurs when a genetic variant is associated with multiple traits, but these traits are on the same causal pathway. While horizontal pleiotropy is problematic for MR, vertical pleiotropy is not. Indeed, if a genetic variant were not associated with all downstream traits on a causal pathway, we may be sceptical about whether the variant truly is mimicking perturbation of the exposure at the head of the pathway. However, the distinction between ‘good’ (vertical) and ‘bad’ (horizontal) pleiotropy can make critical appraisal of studies challenging for readers less familiar with MR.

**Figure 3:**
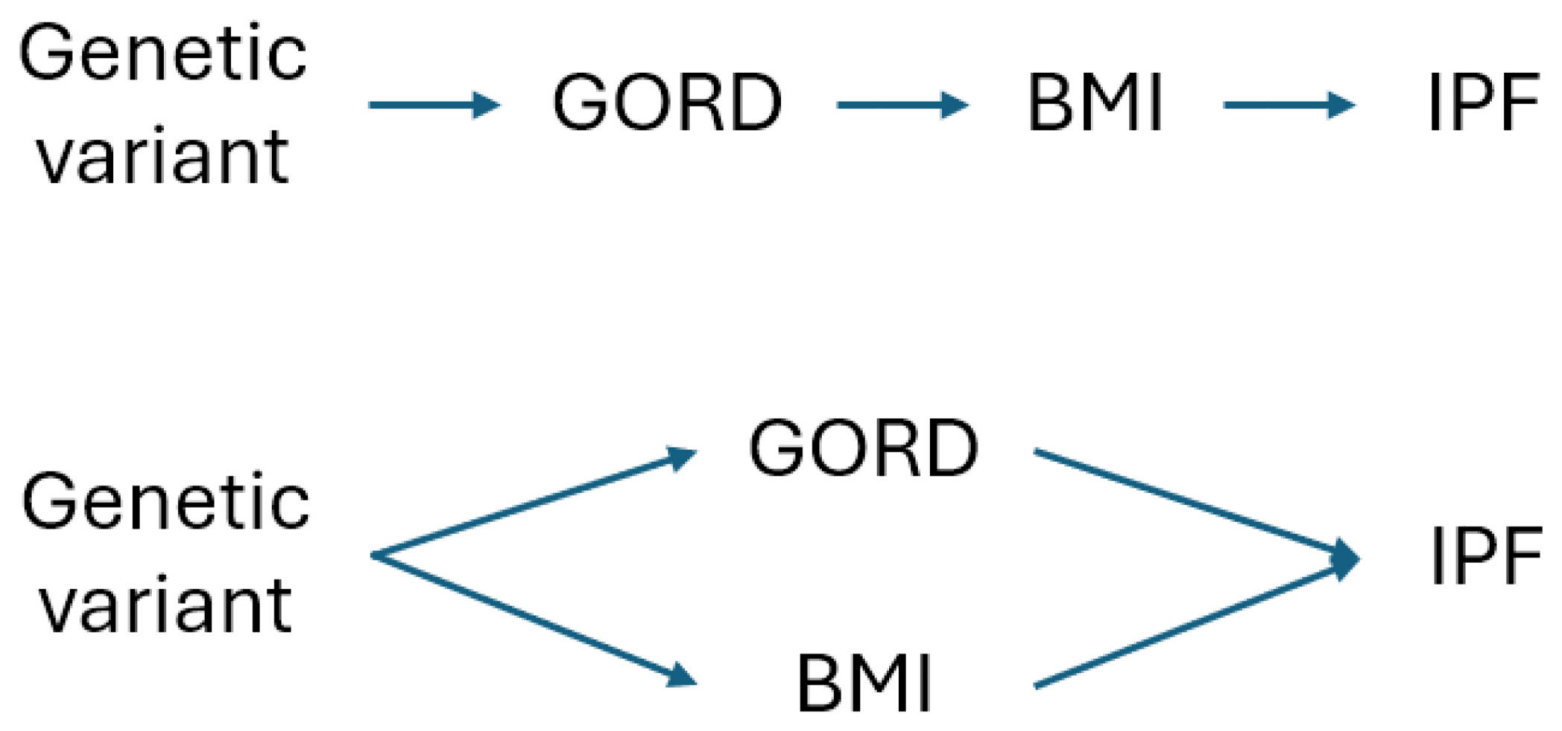
Vertical and horizontal pleiotropy. Illustrative example to distinguish vertical pleiotropy (top panel), which is not a cause for bias, from horizontal pleiotropy (bottom panel), which is. In both diagrams, GORD and BMI are on a causal pathway from the genetic variants to IPF, but the bottom panel represents a violation of a key instrumental variable assumption, as there is a causal pathway from the genetic variants to IPF that does not pass via GORD. Hence, if genetic variants used as instrumental variables for GORD do not affect GORD specifically and primarily, then the MR analysis is not a reliable test of the causal status of GORD. GORD, gastro-oesophageal reflux disease; BMI, body mass index; IPF, idiopathic pulmonary fibrosis.

Take for example a study of the potential effect of gastro-oesophageal reflux disease (GORD) on IPF. GORD and IPF commonly co-exist and microaspiration of gastric acid is hypothesized to contribute to fibrosis. An MR analysis using statistically selected (i.e., biology-agnostic) SNPs to instrument GORD suggested that it increases IPF susceptibility [26]. The claim that MR is more robust against confounding has led some to overlook obvious confounders that would never be ignored in other observational analyses. Obesity and smoking are important risk factors (confounders) for both GORD and IPF [27]. Indeed, some SNPs used to instrument GORD in that study are demonstrably associated with BMI. This is a violation of the third assumption, or horizontal pleiotropy (the variant causing IPF through BMI rather than GORD). However, the authors reassure readers that BMI is on the same causal path as GORD and IPF, therefore such vertical pleiotropy does not bias results [28]. Explicitly specifying the proposed causal pathway would have highlighted this error and helped discern horizontal from vertical pleiotropy. The path for vertical pleiotropy would be if GORD causes elevated BMI which in turn causes IPF. GORD is not an important cause of obesity; rather, the reverse is true [29]. Because causal directions are incorrectly specified, the authors conflate vertical pleiotropy, which is not a cause for bias, with horizontal pleiotropy, which is (Figure 3). This study, which remains published in a leading specialty journal, exemplifies the domain expertise and causal insight required to discern subtle errors. The alleged GORD-IPF association disappears when adjusting for BMI and/or smoking [27].

A purely statistical approach to genetic variant selection risks has an inherent risk of bias. When MR was first proposed two decades ago, genetic variants with known biological function were used to mimic or proxy perturbations in the exposure. By contrast, many MR studies of non-molecular traits apply biology-agnostic selection of genetic variants (i.e., independent, genome-wide significant SNPs) that have no clear mechanistic role. The risk of including variants for alternate causal paths increases as GWAS of the exposure increases in size. For example, larger GWASs of GORD are likely to pick up more genetic variants that have direct effects on upstream risk factors that may influence IPF susceptibility via pathways independent of GORD. Readers should be as cautious about confounding and overconfident causal claims in MR studies as they are with any other observational design. Combining insights from methods that make different assumptions (evidence triangulation) is essential for robust conclusions.

Readers can also increase their confidence in MR findings when results are supported by positive control outcomes. For example, genetically proxied IL-6 receptor inhibition should be able to reproduce established associations with rheumatoid arthritis [30], a licenced indication. Analogously, inconceivable associations with negative control outcomes will highlight potential flaws in the analysis. For example, genetically predicted BMI should not show causal associations with age or sex [31].

### Exposures that share genetic predictors with the outcome

The relationships between different immune mediated inflammatory diseases (IMIDs) are of particular interest to rheumatologists. Many IMIDs co-occur due to shared immune dysregulation, for example, the ‘MHC-I-opathies’ [32] or connective tissue diseases. Having one IMID is also recognised to increases risk of some others; for example, type 1 diabetes and coeliac disease [33]. MR seems an attractive approach to examining potential causal relationships between IMIDs, particularly since residual environmental confounding (e.g., smoking) or reverse causation may be challenging for traditional observational designs. However, analyses of IMIDs as both the exposure and the outcome can be prone to false positives due to shared genetics and pathology. Intuitive examples of why such hypotheses make little sense include examining whether psoriasis causes psoriatic arthritis, or whether axial spondyloarthritis (axSpA) causes uveitis [34], when each pair represents manifestations of shared pathology [35].

Genetic variants within the major histocompatibility complex (MHC) have highly complex linkage disequilibrium with other variants in the region and beyond. For IMIDs, genetic instruments that are correlated with MHC alleles can exhibit large effect sizes and small p-values. If two traits have shared MHC variants, false positives can arise. For example, axSpA and uveitis both have strong associations with HLA-B27 (and other variants in linkage) that can yield significant bidirectional MR associations. Two IMIDs can also be associated with different MHC variants that are in linkage. For example, both lupus and autoimmune hypothyroidism have strong but different MHC associations. Although naïve and pleiotropy robust MR methods both demonstrate strong bidirectional ‘causal’ associations between these two traits, associations disappear when underlying genetic correlation is accounted for [36].

Non-MHC variants that are shared between traits can also produce spurious results. For example, *IL23R* is associated with axSpA, ulcerative colitis and Crohn’s disease. Both naïve and pleiotropy robust MR methods show highly significant bidirectional associations between ulcerative colitis and Crohn’s disease that make no clinical sense and are not present when accounting for shared genetics [36].

When encountering MR analyses of exposure-outcome pairs with potentially shared pathophysiology (e.g., lupus versus celiac disease [37], primary biliary cholangitis [38] or Graves’ disease [39]), readers should consider whether results might be driven by shared genetic predictors rather than a causal effect of one disease on the other. A thorough understanding of the underlying pathology of the traits under investigation is crucial, and papers that fail to demonstrate domain expertise through well-justified hypotheses should be approached with caution.

### IMIDs in biobanks

Genetic data from biobanks are a key driving force behind the explosion in MR papers across all clinical subject areas. Large-scale genetic data are combined with passively collected healthcare data to enable automated GWAS pipelines for every disease with an ICD code. Many rheumatic diseases are rare (e.g., myositis, scleroderma) or prone to misclassification (e.g. axSpA [40]), making MR analyses of biobank data susceptible to bias.

GWAS of rare diseases using biobank data can suffer from severe imbalance in the number of cases and controls. For example, one study tested 302 cases of systemic sclerosis against 213,145 controls [41]. Severe case-control imbalance can produce false positive GWAS hits [42] that, when selected as instrumental variables, bias MR estimates.

Incorrect diagnosis (misclassification) is also a limitation of biobanks. Symptoms such as dry eyes or back pain are common in the general population, and IMID diagnostic codes may appear in health records as differential diagnoses during investigations. Examples such as Sjogren’s and axSpA [40] may be particularly susceptible to misclassification. Many biobank traits are self-reported by participants, which can be an additional source of misclassification. Interpretation of MR findings may be limited unless results can be replicated across different stringencies of case definition.

Biobank data also enable automated MR pipelines that test vast numbers of exposure-outcome pairs, e.g., as implemented through MR Base [43]. It is conceivable that positive results published using biobank data are a result of multiple testing; one suggestive symptom of this is the overwhelming proportion of positive results (i.e., demonstrating a causal relationship) in published MR studies, in contrast to earlier high-profile MR studies that were often null [44,45].

While biobank data have expanded the potential of MR studies, they can also introduce specific biases in the context of IMIDs. Readers should seek validation in samples with independent, preferably criteria-derived case definitions when appraising such studies.

### Reverse causation

MR is said to be more robust against reverse causation because observed traits generally cannot influence germline variants. However, reverse causation can occur when a genetic variant affects the exposure through the outcome. This risk increases as GWAS sample sizes grow and studies of downstream traits pick up upstream genetic associations. For example, MR could be theoretically applied to examine whether rheumatoid arthritis (RA) affects circulating levels of IL-6 signalling cytokines. IL-6 is a key component underpinning RA pathology, that is, the causal effect of IL-6 on RA is well recognised. If variants used to instrument RA liability include those in or correlated with genes involved in IL-6 signalling, then MR estimates showing a causal effect of RA on IL-6 may be due to reverse causation. One way to reduce this risk is to examine the amount of variance explained by the SNP, for example, a variant in the *IL6R* gene should (unless there is greater measurement error) explain greater variance in IL-6R levels than RA. This is the principle behind Steiger filtering [46], a method employed to reduce risk of reverse causation by excluding SNPs that explain more variance in the outcome than exposure. To a rheumatologist, RA affecting IL-6 might seem an obvious example of reverse causation, but more subtle instances can be harder to detect. For example, the effect of RA on IL-6 or CRP as mediators can be estimated when trying to identify causal pathways of RA on cardiovascular risk, in which case it is important to consider whether variants are more likely to instrument RA or the mediator.

### Reasons for optimism

There are good and bad examples of MR as with any study design. The prior explosion in meta-analysis publications was similarly driven by readily available data and software, yet meta-analyses remain a cornerstone of clinical evidence. We therefore conclude optimistically, highlighting some valuable contributions from MR studies.

MR can be used to provide insights into confounding that otherwise would be difficult in traditional epidemiology. Take for example whether statins increase the risk of RA or IPF, or whether beta-blockers increase risk of osteoarthritis (OA) [15,47–49]. Large pharmacoepidemiology analyses that demonstrate an association are unable to fully adjust for important confounders. Data on BMI is often missing in a substantial proportion of the study population (e.g., >60% in the beta-blocker study [47]) and, even if data were complete, BMI is imperfectly measured and (particularly when categorised into obesity/overweight) an imperfect measure of adiposity. Here MR can answer two important questions: first, is there empirical evidence that perturbations in genes mimicking HMGCR or beta-adrenergic receptors are associated with risk of RA, OA or IPF? MR using variants in or near the respective targets of statin [16,50] and beta-blockers [51] do not show an association with these outcomes; therefore, pharmacoepidemiologic associations may not be due to actions of these drugs. Could the pharmacoepidemiologic associations be due to residual confounding? Without complete and accurate data on adiposity, it is difficult to answer this question using traditional observational designs. MR can be used to show that BMI is a common cause of exposures (statin/beta-blocker use) and outcomes with large effect estimates that is likely an important source of residual confounding.

MR can be an important tool for prioritising interventional studies. For example, MR studies of CRP contributed to its de-prioritisation as a therapeutic target for cardiovascular disease [45]. We now know that previously reported associations between CRP and cardiovascular disease were likely driven by IL-6 signalling [52]. MR studies of genetically proxied IL-6 receptor inhibition correctly predicted RCT success for polymyalgia rheumatica [30] and COVID-19 [53]. The same approach was used to inform prioritization of drug targets for sarcoidosis. Although IL-6 signalling is thought to be important in sarcoidosis aetiology, a small clinical trial of IL-6R inhibition showed null preliminary results [54]. The possible futility of larger trials was supported by an MR analysis, which did not provide genetic support that IL-6R inhibition reduces risk of sarcoidosis [55].

Whether adverse effects are related to the underlying disease or the drug can be difficult to disentangle. For example, IL-6R inhibition causes an adverse lipid profile leading to concerns over cardiovascular safety, but RA and associated systemic inflammation also contribute to cardiovascular risk. Neither MR nor an eventual clinical trial showed adverse effects of IL-6R inhibition on cardiovascular risk. In fact, IL-6 inhibition, supported by MR evidence, is being developed as a treatment for cardiovascular disease [52,56].

MR can help assess plausibility of rare adverse drug reactions that would otherwise be challenging or impossible using other study designs. For example, inhibition of the IL-13 pathway for atopic diseases is reported to cause adverse effects that resemble psoriasis or psoriatic arthritis [57]. Whether these adverse effects are causally related to therapy is unclear. Clinical trials, and even studies using large real-world data, are not powered to study ultra rare outcomes. MR supported a causal relationship by showing that genetically proxied IL-13 inhibition is indeed associated with increased risk of psoriatic disease [58]. Another example is the observational association between low serum urate and adverse neuropsychiatric outcomes [59], which is one reason that clinicians are discouraged from excessively lowering serum urate when managing gout. MR findings did not support these concerns [60]. Adverse neuropsychiatric outcomes are not observed in individuals with rare Mendelian causes of hypouricaemia (OMIM 220150), while a clinical trial of urate manipulation was null for Parkinson’s disease [59].

To summarize, MR can help provide insight into sources and magnitude of confounding that may be difficult to achieve in traditional observational analyses with missing data, while well executed drug-target MR is an important tool to assess drug safety and repurposing potential.

## Conclusion

MR-literacy is essential for clinician readers and reviewers faced with the exponential growth in MR publications. Discerning valuable applications relies on a good working knowledge of the biology underpinning hypotheses and what MR can and cannot be used to achieve. Good practices of using reporting checklists, pleiotropy robust sensitivity analyses, and evidence-triangulation can help, but do not replace these sense checks. Specifically, readers should question whether genetic variants can mimic perturbations in the exposure in ways analogous to a proposed intervention, whether the hypothesized causal pathways are plausible, whether results stand up to replication, and whether findings for control outcomes provide supportive evidence. Quality of research can vary substantially using MR as with any design, and all methods have inherent limitations. Readers, equipped with foundational concepts with which to appraise MR analyses, should be better able to discern valuable MR contributions as part of evidence triangulation.

### Practice Points

1. When appraising MR studies, consider whether genetic variants can mimic the exposure in ways analogous to a proposed intervention (gene-environment equivalence).
2. Consider whether the hypothesized causal pathways make sense biologically and clinically.
3. MR findings should be subjected to replication and use of control outcomes, and triangulated with other methodological approaches.

### Research agenda

1. MR analyses to date have mostly been limited to disease incidence as the outcome. Applying MR within a disease population to study secondary outcomes and disease progression remains an important area for research.
2. Drug target MR has primarily leveraged common variant association data. With increasing availability of sequencing data, MR analyses may benefit from using rare variants as instrumental variables.
3. The increasing availability of molecular data presents a great opportunity for targeted MR investigations. However, how to best incorporate different layers of “omics” data is unclear.

## Figures and Tables

**Table 1:** Glossary of key terms.

Relevance assumption	The chosen genetic variant(s) must be robustly associated with the exposure of interest, typically at a genome-wide significance level (p<5x10^-8^).
Independence assumption	The genetic variant must be independent of other factors that affect the outcome. In other words, there must be no confounding pathways between the variant and the outcome.
Exclusion restriction assumption	The genetic variant must only affect the outcome through the exposure under investigation.
Horizontal pleiotropy	When a genetic variant influences the outcome independently of the exposure under investigation, that is, violation of the exclusion restriction assumption. This assumption is the focus of most sensitivity analysis methods (e.g., MR-Egger, weighted median/mode). However, these methods should not be used as a replacement for careful selection of variants (or otherwise application of critical reasoning).
Vertical pleiotropy	When a genetic variant is associated with multiple traits on the same causal pathway to the outcome. This chain of traits to the outcome is what MR sets out to estimate and not a source of bias. For example, a missense variant in the *IL6R* gene influences IL-6 receptor level/function which in turn affects C-reactive protein levels and rheumatoid arthritis.
F statistic	The strength of association between the genetic variant and exposure, and an indicator of the relative (weak instrument) bias that is likely to occur in estimating the exposure-outcome association.
Two-sample MR	MR analysis where gene-exposure and gene-outcome associations are derived from different non-overlapping samples from the same underlying population. Two-sample MR analyses are typically performed using summary level data (e.g., beta and standard error for each genetic variant). The ability to mix and match exposure and outcome genetic association data has made two-sample MR by far the most common method used in MR studies.
One-sample MR	MR analysis using data on the genetic variants, exposure, and outcome from the same sample. One-sample MR analyses are typically performed using individual-level data.
Population stratification	When there are subgroups in the population that have both different phenotypic distributions and different allele frequencies for genetic variants that might be used in MR. This can result in spurious associations between genotype and phenotype.
Assortative mating	When individuals choose their partners non-randomly, for example, taller women tending to partner with taller men.
Dynastic effects	Dynastic or intergenerational effects occur when parental genotype affects offspring outcomes causal pathways independent of the offspring phenotype. For example, when more educated parents support their children’s education.
Linkage disequilibrium	Correlation (non-random association) between genetic variants due to their physical proximity on a chromosome. MR estimates from each genetic variant are pooled analogously to a meta-analysis of clinical trials; therefore, naïvely combining estimates from variants that are not independent (in linkage) is analogous to including a trial in the meta-analysis more than once. Independent genetic variants are often selected by LD ‘clumping’, whereby the most significant variant is selected to represent the locus.
